# Characterization of mesenchymal stem cells in mucolipidosis type II (I-cell disease)

**DOI:** 10.3906/biy-1902-20

**Published:** 2019-06-13

**Authors:** Sevil KÖSE, Fatima AERTS KAYA, Barış KUŞKONMAZ, Duygu UÇKAN ÇETİNKAYA

**Affiliations:** 1 Department of Medical Biology and Genetics, Faculty of Medicine, Atılım University, Ankara, Turkey; 2 Center for Stem Cell Research and Development (PEDI-STEM), Hacettepe University, Ankara, Turkey; 3 Department of Stem Cell Sciences, Graduate School of Health Sciences, Hacettepe University, Ankara, Turkey; 4 BMT Unit, Department of Pediatric Hematology, Hacettepe University Children’s Hospital, Ankara, Turkey

**Keywords:** Mucolipidosis type II, I-cell disease, lysosomal storage disease, bone marrow, mesenchymal stem cells

## Abstract

Mucolipidosis type II (ML-II, I-cell disease) is a fatal inherited lysosomal storage disease caused by a deficiency of the enzyme N-acetylglucosamine-1-phosphotransferase. A characteristic skeletal phenotype is one of the many clinical manifestations of ML-II. Since the mechanisms underlying these skeletal defects in ML-II are not completely understood, we hypothesized that a defect in osteogenic differentiation of ML-II bone marrow mesenchymal stem cells (BM-MSCs) might be responsible for this skeletal phenotype. Here, we assessed and characterized the cellular phenotype of BM-MSCs from a ML-II patient before (BBMT) and after BM transplantation (ABMT), and we compared the results with BM-MSCs from a carrier and a healthy donor. Morphologically, we did not observe differences in ML-II BBMT and ABMT or carrier MSCs in terms of size or granularity. Osteogenic differentiation was not markedly affected by disease or carrier status. Adipogenic differentiation was increased in BBMT ML-II MSCs, but chondrogenic differentiation was decreased in both BBMT and ABMT ML-II MSCs. Immunophenotypically no significant differences were observed between the samples. Interestingly, the proliferative capacity of BBMT and ABMT ML-II MSCs was increased in comparison to MSCs from age-matched healthy donors. These data suggest that MSCs are not likely to cause the skeletal phenotype observed in ML-II, but they may contribute to the pathogenesis of ML-II as a result of lysosomal storage-induced pathology.

## 1. Introduction

Mucolipidosis II (ML-II, MIM# 252500 (McKusick, 2007)) is a rare, autosomal, recessively inherited lysosomal storage disorder, characterized by coarse facial features, progressive psychomotor retardation, severe skeletal abnormalities, growth retardation, thick skin, gingival hyperplasia, hernias, failure to thrive, and death within 5-8 years of age (McKusick, 2007). It is caused by a deficiency of N-acetylglucosamine-1-phosphotransferase (EC 2.7.8.17), which is normally located in the endoplasmic reticulum or Golgi apparatus. The enzyme transfers GlcNAc-phosphate to the oligosaccharide units of lysosomal enzyme precursors (Kudo et al., 2006; Wang et al., 2018). The resulting mannose 6-phosphate (M6P) moiety of these hydrolases normally binds to specific receptors that direct the enzymes to the lysosomes. However, in ML-II, these enzymes are not trafficked properly to the lysosomes and instead leak out of the cells, causing massive accumulation of undigested substrates in lysosomes (Mazrier et al., 2003). These lysosomes have been observed in skin fibroblasts (Otomo et al., 2009), gingival fibroblasts (Koehne et al., 2016), and mesenchymal stromal cells (Leroy et al., 1971; Tondeur et al., 1971) as rough granular cytoplasmic inclusions, hence the origin of the term ‘inclusion cell (I-cell) disease’.Although the molecular basis for this disorder is unknown, evidence indicates that the genetic defect results in an altered oligosaccharide side chain on the I-cell acid hydrolases (Singh et al., 2017). As a result, lysosomal hydrolases can be detected in serum, urine, and cerebrospinal fluid. Heterozygote carriers have been shown to have intermediate levels of phosphotransferase activity. Enzyme replacement therapy (ERT) using a recombinant enzyme, designed to be incorporated by the M6P receptor and directed to lysosomes, is available for some of the lysosomal storage diseases (Schiffmann et al., 2000; Amalfitano et al., 2001; Schiffmann, 2010). However, in the case of ML-II, ERT could not be developed, since the lysosomes display problems trafficking a multitude of enzymes dependent on the M6P receptor pathway. Extrinsic replacement of GlcNAc-phosphotransferase is difficult because of its localization and hexameric composition. Hickman and Neufeld 1972 showed that ML-II fibroblasts could absorb lysosomal enzymes released by normal cells, whereas normal cells could not take up enzymes secreted by ML-II fibroblasts. Hematopoietic stem cells (HSCs) are the source of all mature blood cells and reside in the bone marrow. HSCs have been shown to express and secrete a number of lysosomal enzymes (Lund et al., 2014) and bone marrow transplantation (BMT) has been shown successful in the treatment of leukemias (Wayne et al., 2010), lymphomas (Bhatt and Vose, 2014), anemias (Lucarelli et al., 2012), and certain metabolic disorders (Wynn, 2011). Mesenchymal stem cells (MSCs) are multipotent progenitors that are part of the hematopoietic niche and coexist with HSCs in the bone marrow (BM), but also participate in the regulation of bone mass (Infante and Rodriguez, 2018). MSCs have the ability to differentiate into various cell types, migrate to sites of injury, secrete a wide range of hematopoietic cytokines and growth factors, and have important immune modulatory/immunosuppressive properties. In addition, their ready availability and easy expansion makes them promising candidates for the treatment of many diseases.Since the mechanisms underlying the skeletal defects in ML-II are not completely understood, we hypothesized that the skeletal features of ML-II disease may be partly caused by differentiation defects of MSCs and transplantation of MSCs from healthy donors might be helpful in relieving symptoms caused by the skeletal phenotype. Since transplantation of BM involves coinfusion of both HSCs and MSCs, this formed the basis for considering allogeneic BMT for the treatment of ML-II.Here, we isolated and characterized MSCs isolated from the BM of a ML-II patient before and after BMT and compared the cellular phenotype, based on morphology, proliferation, differentiation, and immunophenotype, with BM-MSCs from a healthy age-matched donor and a ML-II carrier.

## 2. Materials and methods

### 2.1. Patient material

Use of human material was approved by the Hacettepe University Institutional Ethics Committee (FON03/16-3). The ML-II patient was a 4-month-old boy with a homozygous mutation in the *GNPTAB* gene, born to consanguineous parents. The patient presented with a course face, congestive heart failure, hepatosplenomegaly, skeletal deformities, and developmental delay. Based on significantly increased lysosomal enzyme levels of alpha-mannosidase (5233 µmol/L/h), beta-mannosidase (5194 µmol/L/h), beta-hexosaminidase A (678 µmol/l/h), and beta-hexosaminidase A+B (11350 µmol/L/h), the patient was diagnosed with I-cell disease. At the age of 8.5 months, the patient received 4.3 × 106/kg bone marrow CD34+ cells from his HLA identical sibling after myeloablative conditioning with busulfan (16 mg/kg) and cyclophosphamide (200 mg/kg). Mesenchymal stem cells (1 × 106 cell/kg) from the same donor were infused to support hematopoiesis. Peripheral blood chimerism analysis showed 95% donor engraftment at +1 month, 49% at +2 month, and 39% at +3 month posttransplantation. BM samples for MSC analysis were obtained before and at 1 month after transplantation. The carrier used in the study was the sister of the patient. She was 8 years old at the time of bone marrow collection and the presence of a heterozygous mutation in the *GNPTAB* gene was molecularly confirmed. The carrier did not show any specific signs of ML-II. A 1-year-old healthy donor was selected to serve as the nonrelated healthy control. Since this donor was healthy and there was no indication to perform gene screening for a mutation in the *GNPTAB* gene, this was omitted.

### 2.2. Isolation and culture of MSCs

Two milliliters of bone marrow from the ML-II patient, a healthy donor, and a carrier were diluted with PBS and mononuclear cells were isolated using Ficoll Hypaque (1.077 g/L; Biochrom). Cells were cultured in expansion medium consisting of Dulbecco’s modified Eagle’s medium-low glucose (DMEM-LG, GIBCO) supplemented with 10% fetal calf serum (FCS; GIBCO), 35.6% MCDB201 (Sigma), and 1% antibiotics (penicillin 10,000 U/mL, streptomycin 10,000 µg/mL; Biochrom AG). Cells were incubated at 37 °C in 5% CO2 and the medium was changed every 3-4 days. Nonadherent cells were discarded. Analyses were performed using passage 3 (P3) cells.

### 2.3. Differentiation assays

Adipogenic, osteogenic, and chondrogenic differentiation tests were performed to assess the differentiation potential of healthy, carrier, and ML-II BM-MSCs (Köse et al., 2018).

#### 2.3.1. Adipogenic differentiation

MSCs were grown to full confluency on six-well plates and exposed to adipogenic induction medium (DMEM-LG, 10% FCS, 1% penicillin/streptomycin (Biochrom), 1 µM dexamethasone (Sigma), 60 µM indomethacin (Sigma), 0.5 mM isobutylmethyl-xanthine (IBMX; Sigma), and 5 µg/mL insulin (Sigma)) for 21 days. The medium was replaced every 2-3 days. Oil Red O (Sigma-Aldrich) staining was performed for morphologic examination. Semiquantitative measurements were performed by extracting the ORO dye and measuring the absorbance at a wavelength of 492 nm (Tecan).

#### 2.3.2. Osteogenic differentiation

MSCs were cultured for 70% confluency and exposed to osteogenic induction medium (DMEM-LG, 10% FCS, 1% penicillin/streptomycin (Biochrom), 100 nM dexamethasone, 10 mM beta glycerophosphate (Applichem), and 0.2 mM L-ascorbic acid (Sigma)) for 21 days. The medium was replaced every 2-3 days. Cells were stained with Alizarin Red S (Sigma-Aldrich) for morphologic analysis. Semiquantitative analysis of calcium content was performed using a QuantiChrom Calcium Assay Kit (DICA-500, Bioassay systems) and measured at 620 nm.

#### 2.3.3. Chondrogenic differentiation

To confirm the chondrogenic differentiation potential of the MSCs, pellet culture was conducted. Cells were added to 15-mL conical tubes (2 × 105 cells per tube) and centrifuged to form pellets. After 24 h, the culture medium was carefully replaced with serum-free chondrogenic induction medium consisting of DMEM-HG supplemented with 1% penicillin/streptomycin (Biochrom), 10 ng/mL recombinant human TGF-β3 (Merck), 100 nM dexamethasone (Sigma), 0.1 mM L-ascorbic acid (Fluka), 1 mM sodium pyruvate (Sigma), and 1% ITS+ (Collaborative Biomedical Products) in order not to disturb the three-dimensional structure of the small pellets. The medium was changed every 2-3 days for 3 weeks. Quantification was performed using a Blyscan Glycosaminoglycan Assay Kit (Biocolor Life Science) and absorbance was measured at 656 nm.

### 2.4. Immunophenotype of MSCs

P3 MSCs were characterized with respect to the expression of surface antigens. For flow cytometry, cells were trypsinized to obtain a single cell suspension. Cells were incubated for 15 min in the dark at room temperature with 5 µL of fluorochrome-conjugated monoclonal antibody (BD Pharmingen or e-Bioscience). Cells were analyzed for mesenchymal (CD29, CD90, CD105, CD73, CD44, CD49e, and CD146) and other cell surface markers related to immune function or differentiation status of the cells (HLA-DR, HLA-ABC, CD200, ALP, and CD271). After incubation, cells were washed and 10,000 events were recorded for each sample using a FACSARIA flow cytometer (Becton Dickinson, USA). Data were analyzed using BD FACSDiva Software v6.1.2.

### 2.5. Population doubling assay

P3 MSCs were seeded in a six-well plate at a concentration of 3 × 104 cells/well and cultured with expansion medium. Culture medium was replaced every 2 days. Cells were harvested at 3, 5, 7, and 10 days. Viability and cell number were assessed with Trypan blue dye (1:1, v:v).

### 2.6. Statistical analysis 

Descriptive results were presented as mean ± SEM and median (min-max). Normality of the distribution of variables in every study group was evaluated by the Shapiro-Wilk test and differences between study groups (with nonparametric distribution) were assessed by Wilcoxon’s test. P < 0.05 was considered statistically significant.

## 3. Results

### 3.1. Morphology

**Figure 1:**
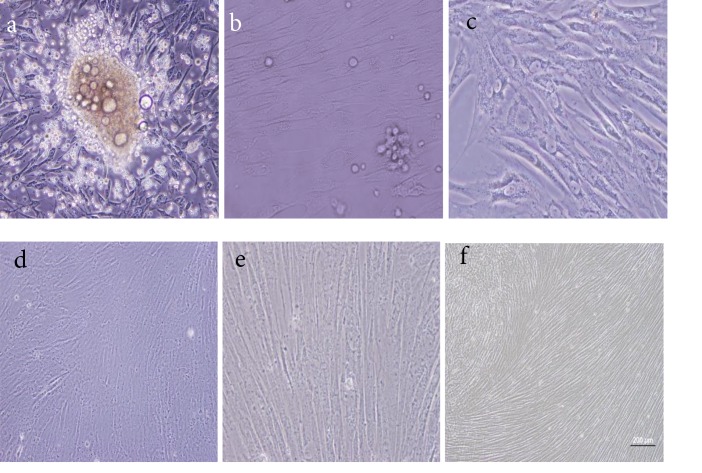
Microscopic analysis of the morphology of human (a, b) BBMT ML-II MSCs (20×, 40×, respectively), (c) ABMT ML-II MSCs (20×), (d, e) carrier MSCs (20×, 40×, respectively), and (f) healthy donor MSCs (10×).

BM-MSCs from all samples showed a spindle-shaped fibroblastic appearance. Intracytoplasmic inclusions were observed in ML-II BM-MSCs both before (BBMT) and after BM transplantation (ABMT) (Figure  1). Hematopoietic cells colocalized with P0 BBMT ML-II BM-MSCs (Figures 1a and 1b), but not with ABMT BM-MSCs or carrier and donor MSCs despite similar culture conditions (Figure  1).

### 3.2. Differentiation capacity of MSCs

**Figure 2:**
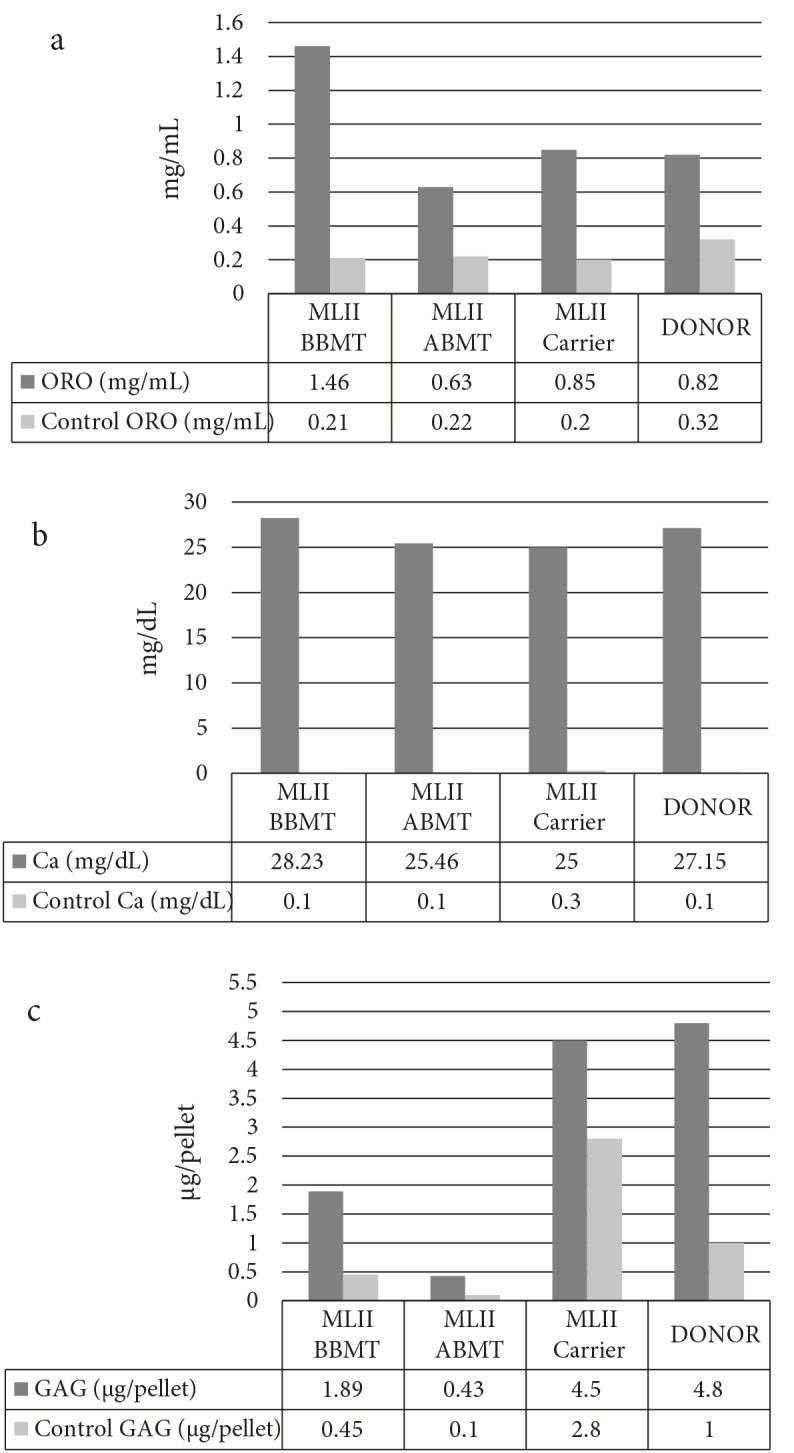
Semiquantitative analysis of adipogenic (a), osteogenic (b), and chondrogenic cell differentiation (c) of human BM-MSCs obtained from MLII BBMT, ML-II ABMT, ML-II carrier, and donor BM.

BM-MSCs from all sources differentiated into adipogenic, osteoblastic, and chondrogenic lineages. Morphologic analyses demonstrated that MSCs retained their ability to form osteoblasts and adipocytes at P3 (Figure  2). The semiquantitative assays for adipogenic, osteogenic, and chondrogenic differentiation showed a better adipogenic differentiation capacity of BBMT ML-II BM-MSCs than ABMT ML-II, carrier ML-II, and donor BM-MSCs (Figure  2a). Conversely, GAG levels were decreased in both BBMT and ABMT ML-II MSCs in comparison to MSC from an age-matched normal control donor and the ML-II carrier. The differentiation potential of BM-MSCs from the ML-II carrier and the unrelated donor was similar.

### 3.3. Immunophenotype of MSCs

Immunophenotyping showed that MSCs from all groups were brightly positive for CD105, CD44, CD73, CD49e, and CD90 and dim positive for CD146, but negative for HLA-DR, HLA-ABC, ALP, CD200, and CD271 surface antigens (Table), confirming the MSC identity of our cells.

**Table:** Cell surface marker percentages of BM-MSCs.

Cell surface marker percentages	ML-II BBMT MSC	ML-II ABMT MSC	ML-II Carrier MSC	Healthy donor MSC
CD44	99.6%	99.3%	99.3%	99.8%
CD73	99.0%	99.4%	99.3%	99.9%
CD105	76.1%	91.3%	84.2%	98.7%
CD146	31.1%	33.1%	16.3%	52.0%
HLA-DR	0.2%	0.1%	3.1%	0.0%
CD49e	99.0%	98.8%	97.7%	99.7%
HLA-ABC	0.5%	0.0%	3.3%	0.1%
CD90	99.6%	98.9%	99.7%	99.6%
ALP	0.0%	0.0%	0.0%	0.6%
CD200	2.3%	0.6%	5.2%	0.1%
CD271	0.0%	0.1%	0.0%	0.3%

### 3.4. Proliferative capacity of MSCs

**Figure 3:**
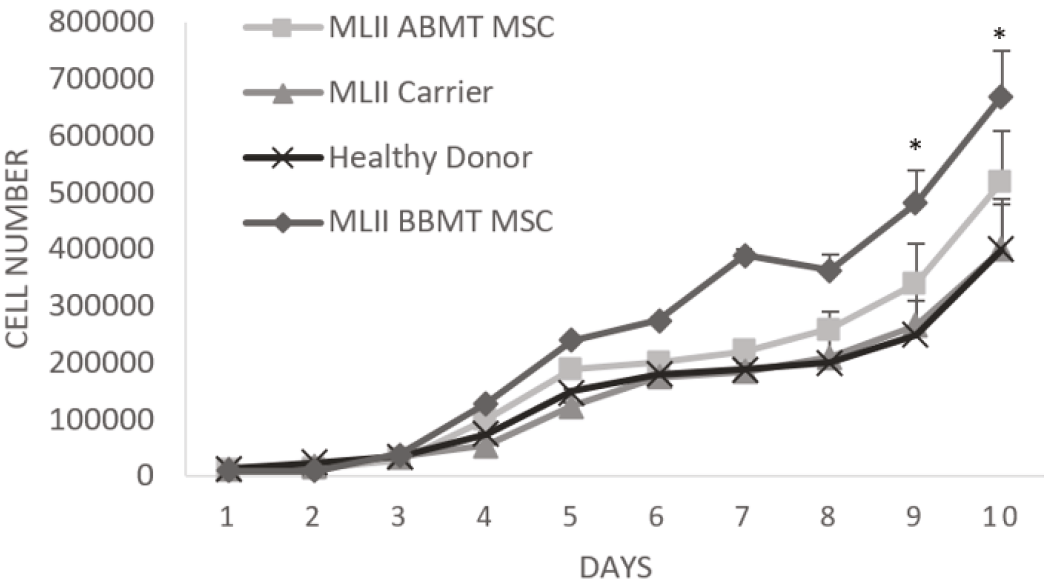
Proliferation of human BBMT, ABMT ML-II, carrier, and healthy donor BM-MSCs (* P < 0.05).

BM-MSC proliferation of the BBMT and ABMT, carrier, and healthy donor samples was similar until day 3. Thereafter, BBMT BM-MSCs showed increased speed of proliferation in comparison to the other groups (Figure  3). This increase became more pronounced on the 9th and 10th days of the analyses compared to other groups and especially healthy donor cells (P < 0.05) and BBMT BM-MSCs (P < 0.05) (Figure  3). 

## 4. Discussion

Mesenchymal stem cells are important components of the BM niche and support maintenance of HSCs through secretion of soluble factors and cell-cell contact (Infante and Rodriguez, 2018). MSCs have been shown to give rise to several different cellular lineages, including osteoblasts (Infante and Rodriguez, 2018). Since the pathophysiology of the skeletal phenotype observed in ML-II has not yet been elucidated, we hypothesized that the differentiation and/or proliferative capacity of ML-II BM-MSCs might be affected and be partially responsible for the bone abnormalities of ML-II patients. Therefore, we compared and characterized the capacities of ML-II patient BM-MSCs before and after BMT with healthy donor and ML-II carrier BM-MSCs.ML-II disease is often called I-cell disease due to the intracellular accumulation of membrane-bound vacuoles in chondrocytes, osteoblasts, osteocytes, and stromal fibroblasts of patients with ML-II (Leroy et al., 1971; Pazzaglia et al., 1992; Poulopoulos et al., 2011). Phase-contrast microscopy of ML-II BBMT and ML-II ABMT BM-MSCs showed accumulation of dense vacuoles or inclusion bodies that are thought to be swollen lysosomes filled with undigested substrates caused by the absence of lysosomal acid hydrolases. Storage materials in lysosomes are likely a mixture of various substrates, since ML-II lysosomes may lack multiple lysosomal enzymes. The colocalization of hematopoietic cells with BBMT ML-II MSCs suggests that they preserve their hematopoiesis-supporting features.Bone changes are a prominent feature of ML-II disease and since the defect in skeletal tissues in ML-II could be caused by abnormal differentiation of BM-MSCs, we compared the differentiation capacity of ABMT, BBMT, carrier, and donor BM-MSCs. While adipogenic differentiation of ABMT and carrier MSCs was similar to that of donor MSCs, BBMT BM-MSCs displayed a higher adipogenic differentiation capacity. This may explain why in certain metabolic and lysosomal storage diseases the fat content of the bone marrow is increased (Tondeur et al., 1982). Increased adipogenic differentiation is also often associated or correlated with a decrease in osteogenic differentiation (Moerman et al., 2004). For instance, it has been observed in humans that when the number of MSCs committed to the adipocyte lineage increases, the number of cells committed to the osteoblast lineage decreases (Maas et al., 2002; Moerman et al., 2004; Pastores and Meere, 2005). Although we did not find such a corresponding decrease in bone differentiation, others have observed a severe reduction of trabecular bone volume associated with an increased cortical porosity in ML-II mice compared to control mice. Interestingly, this phenotype appeared to be the consequence of an unbalanced ratio between osteoblast and osteoclast activities, where bone formation was severely reduced and bone resorption strongly increased (Kollmann et al., 2013). On the other hand, there was no difference in the osteogenic differentiation of BM-MSCs obtained from a patient with Gaucher disease, another lysosomal storage disease (Lecourt et al., 2012).Another possible explanation for the defects in skeletal tissues in ML-II patients could be impaired chondrogenic differentiation of BM-MSCs. The differentiation capacity of BM-MSCs from the carrier was similar to that of healthy donor BM-MSCs, but both BBMT and ABMT BM-MSCs displayed dramatically decreased GAG levels after chondrogenic induction. Skeletal development and homeostasis are coordinated processes that are controlled by three main cell types: chondrocytes in the cartilage and osteoblasts and osteoclasts in bone (Karsenty et al., 2009). During puberty, longitudinal bone growth is mainly regulated by chondrocyte proliferation, differentiation, and matrix deposition, whereas matrix calcification is controlled by the opposed and finely balanced activities of osteoblasts, the bone-forming cells, and osteoclasts, the bone-resorbing cells. Dysregulation of this balance may lead to many severe bone disorders, such as osteopetrosis (Uckan et al., 2009) and osteoporosis (Karsenty et al., 2009). Excessive activity of one of the proteases, cathepsin K, plays a central role in the cartilage morphogenesis defects and type II collagen accumulation observed in ML-II zebrafish embryos (Flanagan-Steet et al., 2009; Petrey et al., 2012) and human blood plasma (Tomatsu et al., 2010). The findings also highlight the fact that activation of secondary biochemical pathways is a common feature of lysosomal diseases.To better understand the pathology of the diseases and/or to develop new treatment targets, further studies should be performed, focusing on in vivo ML-II modeling and assessing the defects at a molecular level. However, before studies with ML-II models in vivo, in vitro immunogenic and inflammatory properties of ML-II MSCs obtained from more patients should be examined in detail. In a study of aspartylglucosaminuria (AGU), a lysosomal storage disease causing neuronal dysfunction and various connective tissue signs, altered collagen and proteoglycan gene expression in cell culture was observed. Reduced mRNA levels of type I and III collagens correlated with the low collagen synthesis in AGU fibroblast cultures (Maatta et al., 1994).We observed that the proliferative capacity of BBMT and ABMT ML-II MSCs was higher than that of carrier and donor cells. Information on the proliferative capacity of fibroblasts or MSCs of this disease is not available in the literature. However, it is known that gingival fibroblasts of the patients show excessive proliferation (Nishimura et al., 2002; Lee and O’Donnell, 2003). Complete loss of cathepsin-L function resulted in the development of gingival overgrowth in these patients (Nishimura et al., 2002). Also, some have suggested that cathepsin D, an aspartic protease, acts as a cell-death mediator (Tardy et al., 2004a). Another explanation might be the relative resistance of I-cell apoptosis induced by cellular stress caused by differences in activity and/or compartmentalization of cathepsins (Terman et al., 2002). Also, TNF-induced cell death was found to be strongly inhibited in I-cell fibroblasts (Tardy et al., 2004b). Another reason for the high apoptosis in cells may be the mitochondria. Mitochondria play key roles in activating apoptosis in mammalian cells (Jeong and Seol, 2008). Mitochondrial dysfunctions in this disorder were shown in human skin fibroblasts (Otomo et al., 2009). However, the study results are limited due to fact that only a single ML-II sample was used. In addition, the age of the patient was very low. In order to prevent bias due to age-related changes, results of the ML-II BM-MSCs were compared to BM-MSCs obtained from a healthy donor of similar age.Allogeneic bone marrow transplantation provides long-term hematopoietic engraftment and is effective in selected lysosomal and peroxisomal storage disorders. Clinical correction of the disease phenotype is variable and based on the organ systems involved, the age of the patient at the time of transplant, and the nature of the metabolic defect. MSCs have the potential to differentiate into cells of bone, cartilage, tendon, muscle, and other adventitial tissues and offer a potential for regenerative tissue therapy. Our challenge is to understand the biology of BM-MSCs and to determine a successful means of their therapeutic use in the clinic. Here, we showed that osteogenic differentiation of ML-II BM-MSCs was not severely affected, although chondrogenic differentiation was somewhat decreased. These differences are unlikely to have a severe effect on the skeletal phenotype; therefore, the skeletal damage observed in ML-II is probably caused by lysosomal storage of multiple enzymes in highly proliferative tissues during embryogenesis and fetal development may be causative. Data from new patients should be used to confirm these results. Lysosomal storage disorders, including ML-II, are excellent candidates for gene therapy since they are well-characterized single-gene disorders and the enzyme expression is generally not subject to complex regulation mechanisms. Although enzyme replacement in these patients is conceptually impossible, isolated MSCs could be genetically modified by lentiviral or CRISPR methodologies to overexpress or correct the mutation in the *GNPTAB* gene. In this way we could get better insight into the precise role of missing gene activity in adipogenesis, osteogenesis, and chondrogenesis and the development of the pathology of the disease. Further studies are needed to better understand the consequences of the genetic defects and the resulting severe skeletal abnormalities observed in ML-II patients.

## Acknowledgment

This study was supported by a grant from the Turkish Ministry for Development, PediSTEM No: 2006-K120640.
